# Use of aromatase inhibitors in practice of gynecology

**DOI:** 10.1186/s13048-015-0131-9

**Published:** 2015-02-25

**Authors:** Betul Usluogullari, Candan Zehra Duvan, Celil Alper Usluogullari

**Affiliations:** Cengiz Gokcek Obstetric and Gynecology State Hospital, Gaziantep, Turkey; Obstetric and Gynecology Department of Turgut Ozal University, Ankara, Turkey; Endocrinology and Metabolism Department of Ersin Arslan State Hospital, Gaziantep, Turkey

## Abstract

**Purpose:**

The conversion of androgens into estrogens by aromatase is called aromatization and is inhibited by aromatase inhibitors (AIs). The aim of this article is to evaluate the use of aromatase inhibitors in gynecological diseases such as endometriosis, leiomyoma, estrogen- dependent gynecologic neoplasia and infertility.

**Methods:**

This is a review of literature combined with experience and use of aromatase ınhıbıtors ın practıce of gynecology.

**Conclusion:**

AIs are promising agents in treatment of estrogen dependent disease. However lack of experience, side effects and cost are limiting factors for using these agents in infertility treatment. However there is need for larger, well designed randomized trials to generate robust data in order to establish the true potential of aromatase inhibitors.

## Introduction

Estrogen is one of the primary steroid hormones for normal female physiology and reproduction. Estrogens (E) are mainly produced in the ovary in a reproductive-age women. Also a small part of E is released from the placental syncytiotrophoblasts, adipose tissue, brain, skin fibroblasts [1].

The three important natural estrogens in women are estrone (E1), estradiol (E2), and estriol (E3). Estradiol is the main form of estrogen in a reproductive-age women.

**Figure 1:**
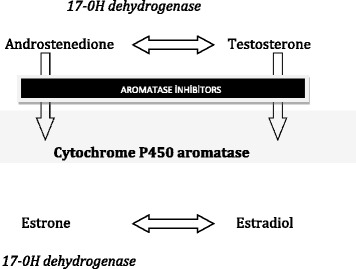
**Aromatase inhibitors block the aromatase cytochrome P450 enzyme.**

Aromatase is a unique enzyme having a crucial role during the synthesis of all estrogens from androgens [2]. The human aromatase enzyme is a member of the cytochrome P450 family and expressed by the CYP19A1 gene located on chromosome 15q21.2 [3,4]. Androstenedione and testosterone are converted to estrogens by the enzyme aromatase. This process called aromatization is inhibited by aromatase inhibitors (AIs) (Figure 1).

As well as in the ovary, aromatase enzyme are available in different tissues such as adipose tissue, liver, muscle, brain, skin, bone, endometrium, and breast tissue. In late 1970s, aromatase inhibitors were used to treat of hormone-dependent breast cancer as an alternative to adrenalectomy [5].

First, aminoglutethimide was a first generation antiepileptic in aromatase inhibitors. But cortisol replacement was needed consequent to CYP11 inhibition by aminoglutethimide. The use of aminoglutethimide was allowed to side effects and the concomitant cortisol need [6].

In the treatment of breast cancer, first selective aromatase inhibitor named as formestan has side effects such as local reactions because of intramuscular administration in clinical practice [7]. This was the trigger in development of other aromatase inhibitors.

AIs can be classified in the below table [8] (Table 1). Nonsteroidal AIs inhibits by competing while a steroidal AI inhibits irreversibly.

**Table 1:** **Classification of aromatase inhibitors**

	**Nonsteroidal Aİ (Reversible)**	**Steroidal Aİ (İrreversible)**
1. Generation	Aminoglutetimid	
2. Generation	Fadrozol	Formestan
3. Generation	Letrozol Anastrozol	Exemestane

Letrozole and anastrazole restrict estrogen levels by 97 to 99% with oral administration at doses of 1 to 5 mg/day [9]. Significant reduction of estrogen in peripheral circulation by the aromatase inhibitors has been promising in the treatment of estrogen-dependent diseases in gynecology practice. Also the use of AIs in early follicular phase for increasing the release of pituitary gonodotropin hormone consequent to estrogen inhibition forces ovulation in infertile patients.

The aim of this review is to evaluate the use of aromatase inhibitors in gynecological diseases such as endometriosis, leiomyoma, estrogen- dependent gynecologic neoplasia and infertility.

### Endometriosis

Endometriosis is a common, benign, chronic and estrogen -dependent disease. characterized by ectopic endometrial glands and stroma. Ectopic endometrial implants is mainly located primarily on pelvic peritoneum and ovaries [10]. Endometriosis affects 5 to 15% of asymptomatic women in reproductive age. In contrast, 30 to 40% of women with infertility have been reported to have endometriosis [11]. Aromatase inhibitors decrease the concentration of circulating estrogens therefore FSH secretion increases. Increased FSH is stimulatory effect on the growth of ovarian follicles in the period of premenopaus [12].

Many factors such as hormonal, enviromental, genetic and defective immune system or cancer may indicted on the pathogenesis of endometriosis. The pathogenesis of endometriosis is not clearly understood and many theories have been put forward. One of important theories for pathogenesis of endometriosis suggests that; during menstruation, there is a reflux of ectopic endometrial tissue into the peritoneal cavity via the fallopian tubes. Studies suggested that endometriotic lesions express aromatase In this way provides local estrogen production in this tissue [13].

Endometriosis demostrate a wide variety of symptoms. The most common findings are infertility and pelvic pain in women. Medical treatment of the disease is to reduce estrogen levels by creating pseudo-menopause and pregnancy status leading to suppression of endometriotic tissue. Treatment aim of endometriosis is to reduce pelvic pain, minimize surgical intervention, and preserve fertility. AIs have been tried in the treatment of symptoms. Aromatase enzime activitiy is located mainly in the ovary and is not detectable in normal endometrium and myometrium [14]. However aromatase enzyme activity and increased expression of the epithelial 17β-hydroxysteroid dehydrogenase type 2 enzyme (17β-HSD2) were demonstrated in the eutopic endometrium from women with endometriosis [15]. These two enzymes are two important steps in the synthesis of estradiol increased estrodiol directly induces prostaglandin synthase-2 (cyclo-oxygenase-2, COX-2), which leads to elevated concentrations of prostoglandin-E2 (PG-E2) in endometriosis. PGE2 in turn, is the most potent known stimulator of aromatase in endometriotic stromal cells. Thus a positive feedback loop occur in the endometriotic tissue for estrodiol synthesis. Ectopic endometrium indicates enhanced ability of proliferation, Inflammation, implantation and angiogenesis through increased estradiol and PGE2 [16]. Aromatase inhibitors effectively suppress estrogen production in the periphery (e.g., brain, adipose tissue) and in endometriotic tissues, as well as in the ovary and decrease circulating estrogen levels considerably. AIs have been successfully used to treat endometriosis-associated pelvic pain. At the studies suggested that effective dose of aromatase inhibitors for the management of pain associated with endometriosis are usually 1 mg daily anastrazole and 2,5 mg daily letrozole [17,18]. At present there are same studies illustrating the efficiency of aromatase inhibitors ın women with pain from rectovaginal endometriosis refractory to other medical or surgical treatment and treating postmenopausal endometriosis [19]. Additionally, AIs significantly reduced the size of the endometrioma to improve pelvic pain in women with ovarian endometriomas as measured by imaging techniques [20].

A systematic review of eight suggested that the evidence of clinical effects of aromatase inhibitors appear to be promising in alleviating pain, reducing lesion size and improving the quality of life in patients with endometriosis. But this systematic review recommended that more powerfull randomised controlled trials are needed [21]. The American College of Obstetricians and Gynecologists (ACOG) committee agrees that AIs are now being used as adjunctive therapy to GnRH analogue in women with endometriosis resistant to conventional medical and surgical therapies. Aromatase inhibitor regimens with add-back progestin or oral contraceptives do not appear to be associated with significant bone loss after 6 months of treatment and may be suitable for long-term use. But randomized controlled trials are needed to compare aromatase inhibitors with traditional medical treatment for endometriosis and to establish the efficacy and side effects of these regimens.

AIs treatment alone increases the risk of ovarian cysts due to induction of ovarian folliculogenesis and can cause bone loss in prolonged use. To avoid this complication in premenopausal women, AIs need to be combined with a progestin, a combination of oral contraceptive, or a GnRH analogue [22]. Unfortunately recurrens of pelvic pain associated with endometriosis is possible after aromatase inhibitors treatment [19].

### Leiomyoma

Uterine leiomyomas are the most common benign tumors of the female genital tract. The clinical incidence is 20-30% at the reproductive age of women. However the true prevalence unknown due to more than one pathologies encountered in pathology specimens. Sex steroids and local growth factors act as the promoter and effector of development of myomas. Aromatase enyzme is upregulated in leiomyoma cells compared with normal myometrium [23]. Increased of aromatase leads to up-regulation of estrogen and progesterone receptors by increasing synthesis of estrogen in the myoma cells. Surgery is the main treatment of symptomatic myoma. However, supression of myoma symptoms by the medical treatment in perimenopausal period prevents stress and complications of surgery until menopause. Aromatase inhibitors create a local hypoestrogenic state abolishing estrogen synthesis in leiomyoma-derived cells and inhibit the proliferation of these cells in uterine myoma [24]. In women in the menopausal transition, AIs reduces the size of uterine leiomyomata and improves symptomatology [25]. The control of the menstrual symptoms in early treatment, even before a marked reduction in uterine volume, shows the extent of the effect of the aromatase inhibitor on the endometrium [26].

AIs are generally better tolerated than many hormonal therapies without the systemic side effects observed with GnRH analogues (phenomenon of “flare up” and menopausal symptoms in general) [27]. Additionally, it does not cause androgenic, progestagenic, or estrogenic effects, such as weight gain, acne, or hypertrichosis, nor does it alter the lipid profile [26]. The production of estrogen and progesterone due to gonadotropin stops and synthesis of androgens from the adrenal gland increases during menopause. Increased androgens are converted to estrogen by the aromatase enzyme in adipose tissue in obese women. Aromatase inhibitors prevent estrogen increase by inhibiting the aromatase enzyme in obese women. Since aromatase inhibitors are comperatively expensive drugs, they can be preferred temporary treatment in women who desire to keep their uterus or who are not suitable for the surgical intervention [28] in women with unexplained infertility with uterine myoma, and also in obese women who fail to respond to GnRHa therapy [29].

### Estrogen- dependent gynecologic neoplasia

Uterine sarcomas are rare forms of uterine malignancy that originate from uterine mesenchymal elements. ER+/PR-(+) endometrial stromal sarcoma should be treated with adjuvant hormonal therapy with aromatase inhibitors for women after total abdominal hysterectomy and bilateral salphingo – oophorectomy [30]. Endometrial hyperplasia is preinvasive neoplastic lesion of endometrial cancer. Aromatase inhibitors are suggested to be used in especially obese women who are diagnosed of atypical or nonatypical endometrial hyperplasia [31]. Aromatase activity was also demonstrated in patients with endometrial cancer. Aromatase inhibitors limit this activity. The studies demonstrated 2–10% of partial response in recurrence endomertrial cancer with aromatase inhibitors [32,33]. Etiologic role of estrogen in ovarian cancer has also been proven [34]. In vitro data suggested that the presence of estrogen receptor in overian tumors is important for treating effect of aromatase inhibitors. Aromatase inhibitors are useful in treatment of estrogen receptor positive ovarian tumors [35].

### Infertility

AI and mainly letrozole provide a new treatment option for ovulation induction in women with anovulatory infertility. They are beneficial in treating unexplained infertility and diminished ovarian reserve. In addition, effects of AIs are being discussed in controlled ovarian hyperstimulation and treatment of infertility related to endometriosis.

### Ovulation induction and controlled overian hyperstimulation

Anovulatory cycles can be seen in 18–25% of women among infertile couples [36]. The most common cause of anovulatory cycles is polycystic ovary syndrome (PCOS) [37]. Clomiphene citrate (CC) is currently the most commonly used pharmacologic agent for ovulation induction in women, and it is approved by FDA in 1967 as the first step of infertility treatment. CC blocks the estrogen receptors centrally and eliminates the negative feedback effect of estrogen on gonadotropins [38]. CC is easy to use and effective in inducing ovulation in most of the patients (60%–90%), but the pregnancy rates are disappointing (10%–40%). This paradox has been attributed to its peripheral antiestrogenic effects, mainly on the endometrium thickness and the cervical mucus [39]. CC treatments are also associated with risk of ovarian hyperstimulation syndrome and multiple pregnancies (10-20%) [40]. CC causes down regulation of estradiol receptors in the pituitary, leading to oversecretion of follicle- stimulating hormone. Oversecretion of this hormone causes formation of multiple follicles and, multiple pregnancies may be seen consequently.

CC resistance can be seen in 20-25% of anovulatory women with PCOS as lack of response to increasing doses of clomiphene citrat [41]. In case of clomiphene citrate resistance, most common step is to switch therapy to injectable gonadotropins. Due to disappointing results with CC treatment, and high price and high risk of complications of gonadotropins (like ovarian hyperstimulation syndrome, painful injection sites), infertility specialists are looking for a new, easy to use, less expensive, and more effective drug like aromatase inhibitors. Letrozole is as effective as CC in ovulation induction [42]. Blocking enzyme production in the ovary by inhibiting aromatization results in an increase in gonodotropin release in early follicular phase. AIs have much shorter half-life (30–60 hours) compered with CC (5 days-3 weeks). Due to rapid weakening effect of aromatase inhibitors in the follicular phase, follicular growth is accompanied by increased estrogen levels. Increase in estradiol allows negative feedback onto hypothalamus and pituitary, because aromatase inhibitors do not deplete estrogen receptors in the brain. Negative feedback leads to decreased gonadotropin levels and atresia of non-dominant follicules, thence increasing the chances of monofollicular ovulation [43]. Initially, aromatase inhibitors have been proposed as effective treatment option for anovulatory women with clomiphene resistance and were used for infertility treatment in anovulatory women in 2001. Oral administration of letrozole, the aromatase inhibitor, was found effective for ovulation induction in CC resistance anovulatory infertil women and endometrial thickness was not affected adversely [44]. Some studies have showed that letrozole use in women resistant to clomiphene can induce ovulation in more patients compared to clomiphene [45]. In AIs treatment, cycles appeared with a better pregnancy rate, probably because of the lack of anti-estrogenic effects of AIs on the endometrium [46] .

Aromatase inhibitors for subfertile women with polycystic ovary syndrome were evaluated by Sebastian Frank et al. in the Cochrane database review in terms of live birth, OHSS, pregnancy, miscarriage and multiple pregnancy. Letrozole appeared to improve live birth and pregnancy rates in subfertile women with anovulatory PCOS, compared to clomiphene citrate. There was not difference in effectiveness between letrozole and laparoscopic ovarian drilling. OHSS could not be evaluated because there was not enough evidence about it [47].

Meta-analyses of six RCTs demonstrated that letrozole improved the ovulation rate per patient; and there was no statistical difference for the ovulation rate per cycle or the pregnancy, live birth, multiple pregnancy or miscarriage rates compared with placebo or with CC plus metformin in women with CC-resistant PCOS [48].

In a study, letrozole is better than laparascopic ovarian drilling (LOD) at six cycles for ovulation rate but difference was not found between letrozole and LOD for pregnancy rate per patient [49]. Postoperative adhesion formation has been documented and there are concerns regarding the effect of LOD on long-term ovarian function [50]. Letrozole seems to be a suitable second-line ovulation-inducing alternative to LOD in women with PCOS who do not conceive with clomiphene citrate. Letrozole is also an effective ovulation inducing agent in women with higher-BMI [51]. In a study comparing occurrence of pregnancy among obese (body mass index: BMI > 30) and nonobese (BMI <30) infertile women (n = 90) with different etiologies, ovulation induction with the aromatase inhibitor letrozole (5 mg on menstrual cycle days 3–7) followed by intrauterine insemination (IUI). As a result, in 90 women undergoing letrozole-IUI treatment showed greater likelihood of pregnancy in higher-BMI group, although the difference was not significant. AIs also are used for the treatment of unexplained infertility. Recent literature supports the use of letrozole in women who appear to be ovulatory, with similar pregnancy rates and fewer pregnancy losses and multiple births compared with CC [52]. In women with unexplained infertility, it appears that letrozole has similar efficacy with injectable gonadotropins when considering ovulation, endometrial thickness, and pregnancy rates, The randomized controlled trials are showing similar pregnancy rates but with significantly reduced costs in the letrozole group when compared with gonadotropins [53]. In a recent study, minimum 40 years of age infertile women (n = 159) undergoing controlled ovarian stimulation and artificial insemination, treated with aromatase inhibitor, letrozole in combination with FSH, exhibited comparable pregnancy rates with less cancelled cycles and less FSH required for stimulation compared to FSH-treated patients alone [54]. In addition, AIs are the promising agent to enhance the follicular response in women with diminished ovarian reserve. Due to inhibition of aromatization, androgens that normally converted to estrogens accumulate in the ovary, and these androgens increase follicular sensitivity to FSH by increasing follicular FSH receptors. The effect of remaining FSH, accelerates follicular development [55]. Also, androgen accumulation in the follicle, stimulates insulin-like growth factor I (IGF-I) which may synergize with FSH to promote folliculogenesis [56]. The improved response was clearly shown by the significantly higher number of mature follicles and significantly lower amount of FSH needed to achieve an adequate number of preovulatory follicles [57]. Further treatment for breast cancer often involves chemotherapy with alkylating agents that can damage ovarian follicle reserve leading to diminished ovarian reserve. For future fertility, oocyte cryopreservation is recommended before chemotherapy. Oktay et al. compared, that use of tamoxifen alone or letrozole combined with low-dose FSH in women with breast cancer who desired embryo cryopreservation. The combination therapy was associated with lower peak E2 levels and a higher number of embryos [58]. Adjunctive use of letrozole may also considered as a cost/effective IVF protocol in these patients [59]. Garcia-Velasco et al. was compared rFSH and highly purified hMG along with antagonist in one group, then added 2.5 mg letrozole to create a second group for comparison. Testosterone and androstenedione were significantly increased in the follicular fluid of the experimental group, compared with the controls. Interestingly, E2 levels of follicular fluid were similar with controls. These findings are consistent with the hypothesis that aromatase inhibition, by blocking androgen to convert into estrogen, increases intraovarian androgens and follicular FSH receptor expression and sensitivity to FSH administration. Also this inhibition can be rapidly reversed. The implantation rate was higher in the letrozole group than gonadotropins alone group (25% and 9.4% respectively). The pregnancy rate was higher (41.6% versus 28.9%) in letrozole group. These two results were not statistically significant [60]. AIs are thought to be useful in treatment of infertility associated with endometriosis. In a recent pilot study, the effect of anastrazole (coupled with goserelin) on endometriomal volume, CA125 levels and standard IVF (in vitro fertilization) fertility parameters were evaluated. This study demonstrated that combined anastrazole and goserelin down-regulation reduces endometriomal volume and serum Ca125 which is in accordance with pregnancy and delivery but a high pregnancy loss was noted [61]. The optimal dose of AIs for ovulation is unclear. The preferred dose of letrozole (2.5) mg or anastrozole (1 mg) in many studies were in similar with doses used for breast cancer treatment in postmenopausal women (generally from days 3 to 7 of menstrual cycle). The first randomized controlled trial addressing letrozole dosing was performed in 2007. Badawy et al. utilized either 2.5, 5, or 7.5 mg for couples with unexplained infertility. Although they found no differences in pregnancy or miscarriage rates, the number of mature follicles was significantly higher in the group of women receiving 7.5 mg daily versus 5 or 2.5 mg [62]. The mechanisms for ovulation induction are similar for letrozole and anastrozole; both AIs are reversible, competitive inhibitors of aromatase, with high potency and receptor selectivity. However letrozole is the preffered one for ovulation induction due to its higher amount of experience about safety and teratogenicity.

### Safety

AIs have been tried for ovulation induction on an off-label basis. Food and Drug Administration (FDA) has categorized the medication as pregnancy category D and the label states that it is contraindicated in women of premenopausal endocrine status, including pregnant women. Initially, there were concerns about letrozole treatment for infertility due to its teratogenic effects. Aromatase inhibitors are not used for ovulation induction in North America, Europe, and other parts of the world due to lack of knowledge about safe dose and effect on fetal toxicity, malformations and outcome. Biljan et al. reported the outcome of 150 babies from 130 pregnancies were compared to a control group of over 36,000 infants born to low risk pregnant women in a community hospital. Although there was no difference in the overall congenital anomaly rate between the two groups, the authors reported that the incidence of cardiac and “bone” anomalies was higher in the letrozole group than in the control group. But in this study the control group was younger (mean age (S.D.) 30.5 ± 1.2 years) than the letrozole group (35.2 ± 4.7 years) [63]. Conversely in another study, incidence of congenital malformations in 911 newborn of women who conceived after treatment with letrazole or clomiphene citrate were compared and no statistically significant difference was observed. Although not statistically significant, increased risk of cardiac anomalies in newborn after ovulation induction with CC was demonstrated [64]. For the half-life of letrazole is shorter than that of CC, letrozole might be effectively cleared from the body by the time of embryo implantation, presumably preventing a teratogenic effect when used in ovulation induction [65]. Complete clearances are in about 10–12 days after the last dose. One study suggests that single dose of 20 mg letrazole on cycle day 3 had the same number of follicles generated and the same pregnancy rate compared with multiple-dose regimen (doses of 5 mg given daily from cycle days 3 to 7). It is hypothesized that administration of a high single dose of AI in the first days of menstrual cycle would achieve maximum estrogen suppression and also rapid clearance of the drug would be possible before the critical final stage of fertilization and embryogenesis[66]. In general, AIs are well tolerated drugs with minimal side effects. The primary adverse effects include menopausal symptoms (vaginal dryness, sexual dysfunction), gastrointestinal events and musculoskeletal symptoms including bone demineralization with high risk of osteoporosis and fracture, arthralgias, and myalgias [67]. Use of aromatase inhibitors are among the major risk factors of osteoporosis. The guidelines recommend that patients who have a T-score > −2.0 but no additional risk factors when AI therapy is initiated should be monitored in every 1–2 years for changes in BMD and risk status. Patients with a T-score < −2.0 at the initiation of AI treatment should receive bisphosphonate therapy [68]. Vitamin D and calcium supplements should be given to all patients receiving AIs. AIs do not increase the risk of endometrial cancer, thromboembolism and cerebrovascular events like tamoxifen [69]. Results of early studies suggest that aromatase inhibitors have adverse effect on the cardiovascular system and lipid profiles compared with tamoxifen. These effects are milder or have not been seen when comparing aromatase inhibitors with placebo but more meticulous study is required in this area.

## Conclusion

AIs are promising agents in treatment of estrogen dependent disease. However lack of experience, side effects and cost are limiting factors for using these agents in infertility treatment. However there is need for larger, well designed randomized trials to generate robust data in order to establish the true potential of aromatase inhibitors.
